# Allergic rhinitis: Incidence and remission from childhood to young adulthood—A prospective study

**DOI:** 10.1111/pai.70078

**Published:** 2025-04-02

**Authors:** Styliana Vasileiadou, Emma Goksör, Göran Wennergren, Eva Rönmark, Linnea Hedman

**Affiliations:** ^1^ Department of Paediatrics, Sahlgrenska Academy University of Gothenburg Gothenburg Sweden; ^2^ Department of Public Health and Clinical Medicine, The OLIN Unit Umeå University Umeå Sweden

**Keywords:** allergic rhinitis, cohort study, incidence, questionnaire, remission, sensitisation

## Abstract

**Background:**

Longitudinal studies on allergic rhinitis (AR) incidence and remission from childhood to adulthood are limited. This study aimed to estimate AR incidence and remission from age 8 to 19 years and to identify related risk factors.

**Methods:**

In 2006, all children in grades 1 and 2 (median age 8 years) in three municipalities in Northern Sweden were invited to participate in a questionnaire survey. The children in two of the municipalities underwent a skin prick test (SPT) for airborne allergens. The protocol was repeated at age 19 years. In total, 2250 participants (91% participation rate) completed the questionnaire, and 1338 underwent SPTs at 8 and 19 years of age.

**Results:**

From age 8 to 19 years, the cumulative incidence of AR was 33.6%, significantly higher among girls than boys (37.4% vs. 29.8%, *p* < .001). Factors that independently increased the risk of developing AR were sensitisation by age 8 (adjusted odds ratio [aOR] 3.75, 95% confidence interval [CI] 2.68–5.23), sensitisation between 8 and 19 years (aOR 2.57, 95% CI 1.82–3.63), and female sex (aOR 1.71, 95% CI 1.30–2.26). The remission rate was 40.0%, with boys experiencing significantly higher remission than girls (45.4% vs. 32.2%, *p* = .015). The probability of remission was decreased by sensitisation before (aOR 0.26, 95% CI 0.13–0.53) and after age 8 years (aOR 0.20, 95% CI 0.05–0.77).

**Conclusion:**

This study found a high incidence of AR between age 8 and 19 years, especially among girls, while boys had a higher remission rate. Sensitisation increased the risk of developing AR and decreased the remission rate.

**Key message**

This study found a high cumulative incidence of allergic rhinitis (AR) from childhood into young adulthood, with girls experiencing higher rates of AR compared to boys. Boys demonstrated significantly higher remission rates, however. Sensitisation before age 8 and during adolescence was strongly associated with an increased risk of AR incidence and reduced odds of AR remission. These findings underline the importance of early sensitisation and intervention in managing AR and improving long‐term outcomes.

## 1 INTRODUCTION

The prevalence of allergic rhinitis (AR) has increased in high‐income countries over recent decades, establishing itself as a leading chronic condition among children and young adults.^1^, ^2^, ^3^, ^4^, ^5^ The negative impact of AR on public health is significant, resulting in impaired sleep, reduced quality of life, decreased school productivity, and less time spent in outdoor activities.^1^

While many cross‐sectional studies have investigated the prevalence of AR, fewer studies have examined the incidence and remission within the same population.^4^, ^6^, ^7^ When assessing incidence, it is essential to repeatedly examine the same study population using consistent methods. Studies of AR in childhood indicate an increasing prevalence with age, suggesting that AR often persists for many years.^4^, ^8^, ^9^, ^10^, ^11^ Sensitisation to airborne allergens is a key factor in the onset of AR, typically preceding the development of the disease.^4^, ^12^, ^13^, ^14^

Although AR is often a chronic condition, clinical remission has been reported in up to 25% of patients.^7^, ^15^, ^16^ Factors associated with remission include the absence of sensitisation,^6^ older age, and absence of baseline asthma symptoms.^7^

The majority of existing research on the progression of AR over time has centered on adult populations,^6^, ^7^, ^16^ with less attention given to children and young adults.^4^, ^15^ Additionally, risk factors associated with the incidence of AR have been sparsely examined.^17^ Understanding prognostic factors related to the incidence and remission of AR is critical for efficient and early healthcare resource allocation. Our primary aim was to estimate AR incidence and remission rates from childhood to young adulthood and to identify risk factors for these outcomes in a population‐based cohort followed from 8 to 19 years of age. Additionally, we investigated the relationship between sensitisation before or after the age of 8 years and the incidence and remission of AR.

## 2 METHODS

### 2.1 Study sample

**FIGURE 1:**
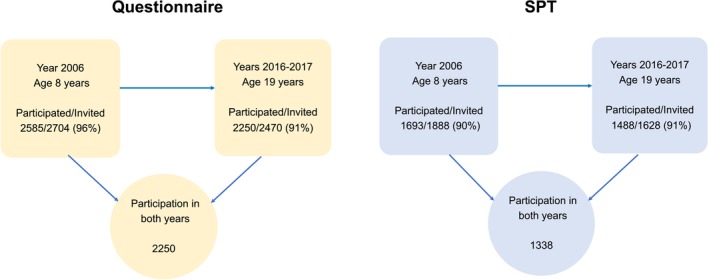
Flowchart illustrating the participation in questionnaire and skin prick test (SPT) at ages 8 and 19 years.

In 2006, as part of the Obstructive Lung Disease in Northern Sweden (OLIN) studies, a cohort study of allergic diseases was initiated. The parents of all schoolchildren in grades one and two (median age 8 years) from three municipalities (Kiruna, Luleå, and Piteå) were invited to a questionnaire survey, yielding a 96% participation rate (*n* = 2585). Children living in Kiruna and Luleå were also invited to undergo a skin prick test (SPT) for common airborne allergens, with 1693 participating (90% of those invited).^18^, ^19^ The cohort was examined using the same methods during the participants' last term of upper secondary school at age 19 years. Of the 2585 participants from 2006, 115 could not be invited to the follow‐up at age 19 for the following reasons: 4 were deceased, 11 could not be traced, and 100 had moved out of the study area. At age 19, 2250 participants completed the questionnaire, resulting in a 91% response rate. In total, 1338 children underwent a SPT at ages 8 and 19 (Figure 1).

The study was approved by the regional ethics review board in Umeå, Sweden. Parental consent was secured for the participants when they were 8 years old, and informed consent was obtained from them at age 19 years.

### 2.2 Questionnaire

The questionnaire comprised the validated International Study of Asthma and Allergies in Childhood (ISAAC) questionnaire,^20^ along with supplementary questions regarding allergic conditions, as thoroughly detailed in previous work.^18^, ^21^, ^22^ The children's parents completed the questionnaire when they were 8 years old and the participants themselves when they were 19 years old.

### 2.3 Allergic sensitisation

Per the European Academy of Allergy and Clinical Immunology recommendations, SPTs were carried out by experienced and specially trained staff. The tests were conducted between February and April in both surveys.^19^, ^23^ The allergens tested were birch, timothy, mugwort, dog, cat, horse, Dermatophagoides farinae, Dermatophagoides pteronyssinus, Cladosporium, and Alternaria (Soluprick, ALK, Hørsholm, Denmark). Positive (histamine 10 mg/mL) and negative controls were incorporated; a positive reaction was defined as a mean wheal diameter of at least 3 mm.

### 2.4 Definitions

The main definitions are presented below. Table S1 presents the definitions of potential risk factors obtained by age 8.

#### 2.4.1 Current symptoms of allergic rhinitis

In the last 12 months, the child has had problems with sneezing, a runny or blocked nose when he/she did not have a cold.^20^

#### 2.4.2 Current medication for allergic rhinitis

In the last 12 months, the child has had to use medication for allergic nose/eye problems.^18^

#### 2.4.3 Current allergic rhinitis

Current symptoms of allergic rhinitis or current medication for allergic rhinitis.

#### 2.4.4 Physician‐diagnosed allergic rhinitis

The child has been diagnosed by a physician as having hay fever or allergic nose/eye problems.^18^

#### 2.4.5 Incidence of allergic rhinitis

The number of new cases of AR between age 8 and 19 years, divided by individuals at risk. Thus, incident cases reported current AR at 19 but not 8 years. The population at risk was children without AR at 8 years of age, excluding those reporting current AR in the first survey. We also considered a stricter population at risk, excluding those with positive responses to any of the questions regarding AR at age 8 years (Table S2).

#### 2.4.6 Remission of allergic rhinitis

Having reported current AR in the questionnaire at age 8 but not at age 19, divided by the number of individuals with current AR at age 8.

#### 2.4.7 Persistent allergic rhinitis

Having reported current AR at ages 8 and 19 years.

#### 2.4.8 Sensitisation

Based on the results of the SPTs performed at age 8 and 19 years, sensitisation was categorised into three groups:

1. No sensitisation: Negative SPT to all tested allergens at 8 and 19 years of age.
2. Sensitisation at age ≤8 years: Positive SPT to at least one tested allergen at 8 years of age.
3. Sensitisation between 8 and 19 years: Negative SPT to all tested allergens at 8 years and positive SPT to at least one tested allergen at 19 years of age.

### 2.5 Data management and statistical analyses

Differences in proportion were analysed by chi‐square tests and risk factor analyses by binary logistic regression, expressed as odds ratios (ORs) with 95% confidence intervals (CIs). Statistical significance was established at *p* < .05.

Analyses of prevalence, incidence, and remission of AR were conducted using data from 2250 children who participated in the survey at ages 8 and 19 years. In analyses including sensitisation, only individuals who underwent SPT at both age 8 and 19 years were included (*n* = 1338). The number of missing answers on individual questions was very low, <3%. The absence of responses to questions about symptoms and diseases was treated as response “no”, as participants were instructed to skip the remaining questions on the disease and not provide further answers if their response to the first question about ever having the disease was “no”. Missing responses about exposures were treated as missing data.

Analyses of potential risk factors for the incidence and remission of AR were based on factors with a *p*‐value <.05 in the unadjusted analysis. Given our focus on early‐life influences on AR, the relevant data were primarily collected from the survey at age 8 years. However, we included SPT results in children at age 8 and 19 years to account for the incidence of sensitisation at these ages.^19^ Regardless of statistical significance, the parents' socioeconomic status (SES) was included in the model due to its potential impact on environmental exposures and lifestyle choices. The adjusted model also included a family history of AR. Associations were expressed as adjusted ORs (aORs) with 95% CIs.

All statistical analyses were performed using IBM SPSS Statistics, version 29 (IBM Corporation, Armonk, NY, U.S.A.).

## 3 RESULTS

### 3.1 Νon‐responders

We analysed the differences between children participating at ages 8 and 19 years (responders) and those who participated only at age 8 but not at 19 years (non‐responders). The non‐responders were less likely to have a family history of AR and more likely to have been exposed to parental smoking during the first year of life. There was also a higher prevalence of manual workers and fewer non‐manual employees among parents of the non‐responders (Table S3).

### 3.2 Prevalence of AR

The prevalence of allergic symptoms, medication use, physician diagnoses, and allergic sensitisation is detailed in Table 1. The prevalence of current AR increased from 15.6% (350/2250) among 8‐year‐olds to 37.7% (849/2250) among 19‐year‐olds, *p* < .001. At 8 years of age, the prevalence of AR was higher in boys (18.0%, 207/1153) than in girls (13.0%, 143/1097), *p* = .001. However, a notable shift emerged at age 19, where the prevalence of AR was higher in girls (41.4%, 454/1097) than in boys (34.3%, 395/1153), *p* < .001 (Table 1).

**TABLE 1:** The prevalence of symptoms and use of medicine for allergic rhinitis (AR), current AR, and physician‐diagnosed AR, asthma, or eczema among 2250 individuals that participated at ages 8 and 19 years. Allergic sensitisation was also assessed via skin prick tests in a subset of 1338 participants at ages 8 and 19 years. Data were analysed for the entire sample and stratified by sex.

Symptoms and diseases	2006				2016–2017			
	Total % (*n* = 2250)	Boys % (*n* = 1153)	Girls % (*n* = 1097)	Difference by sex, *p*‐value	Total % (*n* = 2250)	Boys % (*n* = 1153)	Girls % (*n* = 1097)	Difference by sex, *p*‐value
Current AR symptoms	15.1 (339)	17.5 (202)	12.5 (137)	<.001	35.6 (800)	31.7 (366)	39.6 (434)	<.001
Current AR medication	10.2 (229)	11.4 (132)	8.8 (97)	.043	22.2 (499)	18.9 (218)	25.6 (281)	<.001
Current AR	15.6 (350)	18.0 (207)	13.0 (143)	.001	37.7 (849)	34.3 (395)	41.4 (454)	<.001
Physician‐diagnosed AR	7.8 (175)	8.9 (103)	6.6 (72)	.040	8.9 (201)	8.1 (93)	9.8 (108)	.140
Physician‐diagnosed asthma	7.1 (160)	9.1 (105)	5.0 (55)	<.001	17.6 (395)	17.6 (203)	17.5 (192)	.956
Physician‐diagnosed eczema	15.4 (346)	16.4 (189)	14.3 (157)	.179	14.7 (331)	11.6 (134)	18.0 (197)	<.001
Sensitisation								
At least one positive skin prick test	30.9 (413/1338)	34.3 (244/654)	27.6 (189/684)	.009	46.8 (626/1338)	50.8 (332/654)	43.0 (294/684)	.004

Abbreviation: AR, allergic rhinitis.

The prevalence of physician‐diagnosed AR at age 8 was 7.8% (175/2250) compared to 8.9% (201/2250) at age 19, *p* < .001. Among individuals with current AR at 8 years, 39.1% (137/350) reported physician‐diagnosed AR and 60.3% (211/350) reported allergic conjunctivitis. At 19 years of age, 19.7% (167/849) of those with current AR reported a physician's diagnosis of AR and 52.9% (449/849) reported allergic conjunctivitis.

### 3.3 Incidence of AR

Based on the population at risk defined by the absence of current AR at age 8, the incidence of AR by age 19 was 33.6% (639/1900, Figure 2), representing an average annual incidence of 3%. The incidence rate did not differ even when employing a more stringent definition of the at‐risk population, excluding those who answered affirmatively to any of the questions about AR at 8 years (32.7%, 590/1807). The incidence of AR was significantly higher among girls (37.4%, 357/954) than boys (29.8%, 282/946), *p* < .001.

**FIGURE 2:**
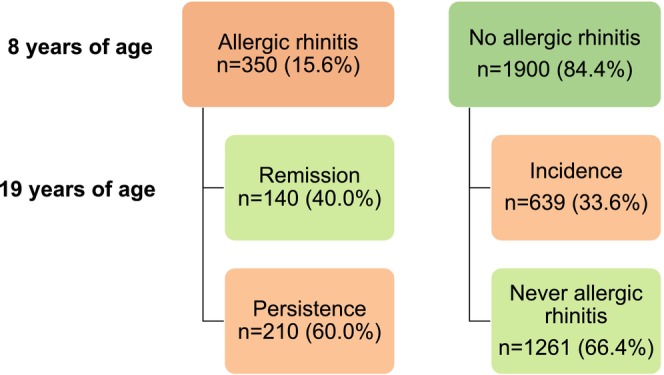
The number (%) of individuals with or without allergic rhinitis at 8 years of age and those without allergic rhinitis, incidence, remission, and persistence of allergic rhinitis at 19 years.

### 3.4 Incidence of AR in relation to sensitisation

By age 8, 30.9% (413/1338) of participants exhibited sensitisation; between ages 8 and 19, this figure was 17.7% (237/1338); and 51.4% (688/1338) had negative tests on both occasions. Considering only participants without AR at age 8 who subsequently underwent SPT at ages 8 and 19, the incidence of AR was 37.1% (415/1120).

Among those with incident AR, 35.4% (147/415) were sensitised by age 8; 24.6% (102/415) between ages 8 and 19 years; and 40.0% (166/415) showed no sensitisation. Among participants sensitised by age 8, the incidence of AR was 55.7% (147/264); among those sensitised between ages 8 and 19, it was 46.4% (102/220); and among those without sensitisation, it was 26.1% (166/636).

### 3.5 Factors associated with AR incidence

The potential risk factors for AR incidence, assessed through unadjusted analyses, are outlined in Table 2. In the unadjusted analyses, statistically significant factors included female sex, a family history of AR, consuming fast food at least once a week, and a history of eczema, asthma, or food allergy at age 8. Both sensitisation by age 8 and between ages 8 and 19 years increased the risk of incident AR, whereas parental smoking during the first year of life showed a protective effect.

**TABLE 2:** Factors investigated for their association with the cumulative incidence of allergic rhinitis (AR) from age 8 to 19 years, expressed as unadjusted odds ratios with 95% confidence intervals.

Factors	Prevalence of factors among all % (*n* = 2250)	Cumulative incidence of AR % (*n* = 639)	*p*‐Value	OR	95% CI
Sex					
Male	51.2 (1153/2250)	29.8 (282/946)		1	Ref.
Female	48.8 (1097/2250)	37.4 (357/954)	<.001	1.41	1.16–1.71
Family history of AR					
No	56.1 (1263/2250)	30.8 (352/1144)		1	Ref.
Yes	43.9 (987/2250)	38.0 (287/756)	.001	1.38	1.14–1.67
Parental smoking the first year of life					
No	81.8 (1798/2199)	34.7 (527/1517)		1	Ref.
Yes	18.2 (401/2199)	28.7 (97/338)	.034	0.76	0.58–0.98
Fast food at least once a week					
No	89.5 (2003/2239)	32.5 (549/1690)			
Yes	10.5 (236/2239)	42.8 (86/201)	.004	1.55	1.15–2.09
Socioeconomic status					
Manual workers	25.0 (517/2065)	34.7 (158/455)	.900	1	Ref.
Unemployed	1.4 (28/2065)	14.3 (3/21)		0.31	0.09–1.08
Non‐manual employees	44.0 (908/2065)	32.7 (247/755)		0.91	0.72–1.17
Self‐employed	6.3 (130/2065)	36.1 (39/108)		1.06	0.69–1.65
Professionals and executives	23.3 (482/2065)	33.1 (131/396)		0.93	0.70–1.24
Eczema at age 8 years					
No	84.6 (1904/2250)	32.3 (537/1665)			
Yes	15.4 (346/2250)	43.4 (102/235)	<.001	1.61	1.22–2.13
Asthma at age 8 years					
No	92.9 (2090/2250)	33.0 (598/1811)			
Yes	7.1 (160/2250)	46.1 (41/89)	.015	1.73	1.13–2.66
Food allergy at age 8 years					
No	86.4 (1944/2250)	32.6 (553/1694)			
Yes	13.6 (306/2250)	41.7 (86/206)	.010	1.48	1.10–1.99
Sensitisation					
No sensitisation	51.4 (688/1338)	26.1 (166/636)	<.001	1	Ref.
Sensitisation at age ≤8 years	30.9 (413/1338)	55.7 (147/264)		3.56	2.63–4.80
Sensitisation at age >8 years	17.7 (237/1338)	46.4 (102/220)		2.45	1.78–3.37
Short breastfeeding (<4 months)					
No	14.5 (314/2170)	31.3 (85/272)		1	Ref.
Yes	85.5 (1856/2170)	34.0 (529/1557)	.404	1.13	0.86–1.49
Maternal smoking in pregnancy					
No	90.5 (2004/2215)	34.0 (577/1698)		1	Ref.
Yes	9.5 (211/2215)	30.6 (53/173)	.399	0.86	0.61–1.20
Number of siblings					
0	6.9 (152/2210)	36.1 (44/122)	.844	1	Ref.
1	51.3 (1134/2210)	34.3 (328/957)		0.92	0.62–1.37
2	26.9 (595/2210)	29.9 (153/511)		0.76	0.50–1.15
≥3	14.9 (329/2210)	38.5 (106/275)		1.11	0.72–1.73
Area of living the first year of life					
Urban	63.0 (1342/2129)	32.5 (366/1125)	.258	1	Ref.
Rural	37.0 (787/2129)	35.2 (239/679)		1.13	0.92–1.38
Ever living on a farm					
No	97.4 (2178/2235)	33.6 (617/1837)	1.000	1	Ref.
Yes	2.6 (57/2235)	32.7 (17/52)		0.96	0.53–1.73
Heavy traffic road close to home					
No	58.7 (1291/2200)	32.8 (362/1105)			
Yes	41.3 (909/2200)	34.3 (258/752)	.515	1.07	0.88–1.30
Dampness at home					
No	88.5 (1943/2196)	33.7 (554/1644)			
Yes	11.5 (253/2196)	32.1 (68/212)	.699	0.93	0.68–1.26
Ever cat at home					
No	71.6 (1587/2215)	33.9 (446/1316)			
Yes	28.4 (628/2215)	33.2 (185/557)	.789	0.97	0.79–1.20
Ever dog at home					
No	63.8 (1411/2213)	33.8 (397/1173)			
Yes	36.2 (802/2213)	33.5 (234/698)	.919	0.97	0.81–1.20
Parental smoking					
No	78.5 (1735/2210)	34.0 (499/1467)			
Yes	21.5 (475/2210)	32.0 (128/400)	.474	0.91	0.72–1.16
Fish less than once per week					
No	82.3 (1835/2231)	34.4 (536/1558)			
Yes	17.7 (396/2231)	30.0 (98/327)	.139	0.82	0.63–1.06
Fruit less than every day					
No	78.1 (1746/2236)	34.5 (511/1481)			
Yes	21.9 (490/2236)	30.6 (125/408)	.156	0.84	0.66–1.06

Abbreviations: 95% CI, 95% confidence interval; AR, allergic rhinitis; OR, odds ratio.

**FIGURE 3:**
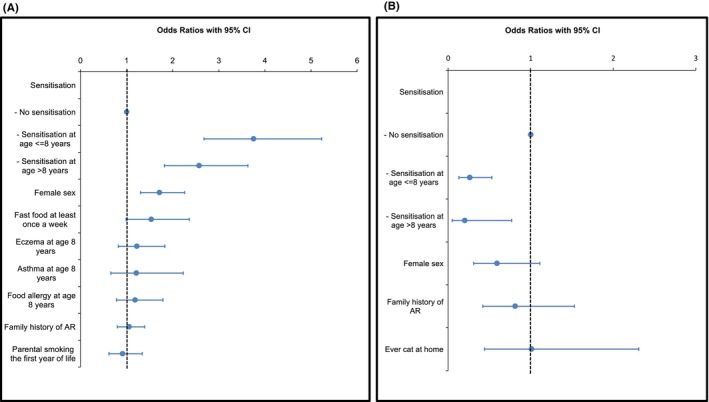
Forest plots illustrating the factors included in the adjusted analysis of the incidence (A) and remission (B) of allergic rhinitis (AR). The analysis was also adjusted for parental socioeconomic status.

In the adjusted logistic regression analysis, factors associated with the incidence of AR were sensitisation by age 8, sensitisation between ages 8 and 19 years, and female sex (Figure 3A and Table S4). In this model, among the atopic conditions of sensitisation, asthma, eczema, and food allergy at age 8, only sensitisation reached statistical significance. However, when these atopic conditions were included one at a time, sensitisation and eczema reached significance, while asthma and food allergy did not (Table S5).

After stratification by sensitisation status, female sex remained an independent risk factor for the incidence of AR, both for non‐sensitised (aOR 1.61, 95% CI 1.08–2.38) and sensitised group by age 19 (aOR 1.82, 95% CI 1.23–2.68). No additional factors achieved statistical significance in either group.

### 3.6 Remission and AR persistence

At age 19 years, the remission rate of AR was 40.0% (140/350, Figure 2) and was significantly higher for boys (45.4%, 94/207) than for girls (32.2%, 46/143), *p* = .015. Of those with persistent AR, 140 of 210 (66.7%) experienced symptoms impacting their daily activities at age 19.

### 3.7 Remission of AR in relation to sensitisation

Among participants who underwent SPT testing at both 8 and 19 years, the rate of AR remission was 37.6% (82/218). Of those in remission, 54.9% (45/82) were sensitised by age 8, 4.9% (4/82) between the ages 8 and 19, and 40.2% (33/82) showed no sensitisation. Of those in remission, 54.9% (45/82) were found to be sensitised at the age of 19 years (41 still sensitised from 8 years and 4 not sensitised at 8 years but sensitised at 19 years). The remission rate was 63.5% (33/52) among those with current AR but without sensitisation at 8 years. In comparison, the remission rate was 30.2% (45/149) in those sensitised by age 8 and 23.5% (4/17) in those sensitised between the ages 8 and 19.

### 3.8 Factors associated with AR remission

Unadjusted analyses revealed that female sex, sensitisation by age 8, and sensitisation between ages 8 and 19 were associated with decreased odds of achieving AR remission by age 19. Conversely, having a cat at home and the SES of professionals/executives increased the odds of remission and constituted a protective factor (Table 3). Factors tested but found not to be significant included family history of AR, short breastfeeding (<4 months), maternal smoking in pregnancy, parental smoking in the first year of life, the number of siblings, area of living in the first year of life (urban/rural), ever living on a farm, heavy traffic road close to home, dampness at home, having a dog at home, parental smoking, eating fish less than once/week, fruit less than every day, fast food at least once/week, and having eczema, asthma, or food allergy at 8 years of age (Table S6).

**TABLE 3:** Factors associated with remission of allergic rhinitis (AR) from age 8 to 19 years, expressed as unadjusted odds ratios with 95% confidence intervals.

Factors	Remission of AR % (*n* = 140)	*p*‐Value	OR	95% CI
Sex				
Male	45.4 (94/207)		1	Ref.
Female	32.2 (46/143)	.015	0.57	0.37–0.89
Ever cat at home				
No	37.3 (101/271)		1	Ref.
Yes	50.7 (36/71)	.042	1.73	1.02–2.93
Socioeconomic status				
Manual workers	30.6 (19/62)	.078	1	Ref.
Unemployed	28.6 (2/7)		0.91	0.16–5.09
Non‐manual employees	41.2 (63/153)		1.58	0.85–2.97
Self‐employed	22.7 (5/22)		0.67	0.21–2.07
Professionals and executives	47.7 (41/86)		2.06	1.04–4.10
Sensitisation				
No sensitisation	63.5 (33/52)	<.001	1	Ref.
Sensitisation at age ≤8 years	30.2 (45/149)		0.25	0.13–0.48
Sensitisation at age >8 years	23.5 (4/17)		0.18	0.05–0.62

Abbreviation: AR, Allergic rhinitis.

In the adjusted logistic regression analysis, sensitisation by age 8 and sensitisation between ages 8 and 19 decreased the odds of achieving AR remission (Figure 3B and Table S7). When the same multivariate logistic regression model was stratified by sensitisation into no sensitisation and sensitisation up to age 19, the SES of professionals/executives increased the odds of remission (aOR 3.45, 95% CI 1.12–10.67) among those sensitised. Conversely, none of the factors in the multivariate logistic regression reached statistical significance in the non‐sensitised group.

## 4 DISCUSSION

The main findings are the significantly higher incidence of AR among females and the higher remission rate among males. Both sensitisation by age 8 and sensitisation between age 8 and 19 years increased the risk of incident AR and lowered the odds of remission. Approximately 50% of the individuals with sensitisation had developed AR by age 19 compared to only one in four among those not sensitised.

Our study provides valuable insights into AR progression during childhood and adolescence, highlighting the notable sex differences in incidence and remission rates. A higher incidence of AR was observed in girls, while boys exhibited a greater remission rate by age 19. These findings align with previous studies, suggesting age‐dependent sex differences in AR incidence.^17^, ^24^ A systematic review and meta‐analysis identified a male predominance of AR in childhood, transitioning to female predominance in adolescence, potentially attributable to hormonal changes.^24^

This transition suggests a divergence in the impact of puberty on immune responses and AR susceptibility between the sexes. Sex differences are less evident in adult study populations. In the large population‐based RHINE study, no statistically significant sex differences in the clinical remission of AR in adults were found.^16^

The cumulative incidence of AR in our cohort between ages 8 and 19 years reached 33.6%, representing an average annual incidence of 3%. This finding is in line with other Nordic and European studies reporting an incidence of about 1–3% per year.^4^, ^25^, ^26^ For example, the Swedish BAMSE study reported an average estimated annual incidence of 1.5% between 4 and 8 years, 1.3% between 8 and 16 years, and 0.8% between 16 and 24 years.^4^ Similarly, an American study observed a 17.2% incidence of rhinitis during the first 5 years of life, translating to an average of 3.4% per year.^25^ Moreover, a Swedish adult cohort aged 20–59 reported a peak incidence of 8.7% among those aged 20–29, approximating an annual rate of 1%, with a subsequent decline observed in older age groups.^7^ Thus, the elevated prevalence and incidence of AR are particularly pronounced during childhood and adolescence, underscoring the need for early detection and intervention.

Our study also revealed that sensitisation by age 8 was a strong predictor of AR, consistent with previous research, including the Swedish BAMSE study, which demonstrated that childhood sensitisation to inhaled allergens frequently preceded AR symptoms.^4^ Similarly, another recently published Swedish study reported that the majority (84%) of individuals sensitised by age 8 developed asthma or rhinitis at 19 years of age,^12^ further strengthening the view that early sensitisation significantly increases the risk of AR development.^4^, ^27^ Conversely, other studies suggest that rhinitis symptoms may appear before sensitisation,^28^, ^29^, ^30^ indicating the complex nature of AR development. Approximately 25% of the individuals with incident AR in our cohort developed sensitisation between the ages of 8 and 19; the temporal precedence of sensitisation versus rhinitis symptoms in this subgroup remains undetermined.

The stronger association with sensitisation by 8 years compared to sensitisation between 8 and 19 years suggests a causal link between sensitisation and AR. However, this does not preclude a reverse relationship. Further research is needed to elucidate this relationship.

Among participants in our study, approximately 40.0% of those with AR at age 8 achieved remission by age 19; this outcome was most prevalent in the non‐sensitised group. In addition, more than half of those in remission were found to be sensitised at 19 years, suggesting that remission was not necessarily associated with a loss of sensitisation despite symptom resolution. This high remission rate is comparable to that observed in other European cohorts (e.g., the RHINE study), where participants were followed longitudinally from ages 20–44 at baseline to ages 30–54 and later 38–66 years. A clinical remission rate of 31.8% was reported, with remission being more common among non‐sensitised individuals, often occurring later in life.^16^ Similarly, a Danish study, which included participants aged 15–69 in 1990 and followed up in 1998, reported remission rates of 12% for pollen AR, 19% for animal AR, and 38% for house dust mite AR; remission was associated with low IgE levels.^15^ These findings support a higher probability of achieving AR remission among non‐sensitised individuals.

On the other hand, some studies have reported lower remission rates than ours. For instance, another Swedish study found a 23.1% remission rate between 1992 and 2000 among adults aged 20–59 years at baseline.^7^ Similarly, the BAMSE study reported lower remission rates, particularly between the ages of 16 and 24 years.^4^

While our study identified female sex and sensitisation as significant risk factors for AR incidence, other factors previously identified as significant did not reach significance in our cohort. For example, although family history of allergic conditions is known to increase the risk of AR, suggesting a hereditary predisposition, we failed to observe a significant effect of family history on disease incidence or remission outcomes.^7^, ^17^ Family history of AR was a significant risk factor for AR incidence in the unadjusted analysis and remained significant in the adjusted analysis. However, when sensitisation was added to the adjusted analysis, the significance of family history disappeared, possibly due to sensitisation being a primary risk factor. Similarly, while asthma, eczema, and food allergy were significant risk factors for AR incidence in the unadjusted analysis, they did not reach significance in the adjusted analysis. When they were added in the adjusted analysis one at a time, sensitisation and eczema reached significance.

Individuals raised in rural settings or on farms exhibit a documented protective effect, likely due to early allergen exposure fostering enhanced immunological tolerance.^5^, ^31^, ^32^ However, our results did not show a significant association between rural or farm exposure and AR incidence or remission. One explanation for this might be the small number of participants who reported living on a farm at 8 years of age. For this reason, we also analysed the area of living (urban/rural), but it did not reach significance either, as a rural environment may not be equivalent to a farm environment with farm animals, animal sheds, and similar exposures. Additionally, the three municipalities involved in the study are small towns close to nature, making it difficult to distinguish between urban and rural environments.

While some studies suggest a link between parental smoking (particularly maternal smoking during pregnancy or early childhood) and an elevated risk of AR,^17^ our findings revealed an inverse association between parental smoking during the first year of life and AR incidence in the unadjusted analysis; however, this association did not reach significance in the adjusted analysis.

In three cohorts of children participating in the OLIN studies, no association was detected between maternal smoking and allergic sensitisation. However, a trend suggested a protective effect but did not reach statistical significance.^33^ In the West Sweden Asthma Study, an inverse dose‐dependent relationship between smoking and the prevalence of AR was observed. This negative association was particularly evident in men, whereas no such pattern emerged among women.^34^ Similarly, smoking was associated with a higher likelihood of AR remission in the RHINE study.^16^

A potential mechanism that could explain the “protective effect” of parental smoking exposure or smoking in adulthood on AR is immune system modulation.^35^ Smoking may have a strong immunosuppressive effect that downregulates certain allergic pathways, thereby reducing inflammation in response to allergens.^35^, ^36^ Furthermore, tobacco exposure might trigger epigenetic modifications that affect immune function in offspring,^3^ potentially altering their susceptibility to allergies. Finally, the supposed “protective effect” might be due to selection bias or other environmental influences.

This study has several strengths, including a large sample size, high participation rates, and a longitudinal design. Our sample is representative of the population, comprising an unselected cohort with a high participation rate. Consequently, the risk of selection bias is minimal. Moreover, as 90% of the original cohort participated in the survey at 19 years, the risk of attrition bias is low. Data from two observation points, collected over an 11‐year period, allowed for a thorough analysis of AR incidence and remission, encompassing the frequently gradual progression and regression of clinical symptoms. Cross‐sectional studies often miss such data. A select team of trained specialists conducted all SPTs, using standardized test extracts across all surveys. Allergic sensitisation was mainly assessed through SPTs, demonstrating good agreement with specific IgE testing in our cohort^19^ and other studies.^37^

This study has two key limitations: the self‐reported nature of the AR variable, which remains unvalidated, and the absence of data on AR triggers. However, the ISAAC questions used in this AR study have been validated in previous research.^38^ Our definition of current AR included current medication use or the presence of current symptoms, and current AR was used to build the definition of AR incidence and remission. We did not choose to rely on a physician's diagnosis because many children in Sweden use over‐the‐counter medications to manage AR symptoms without seeking medical consultation and a diagnosis by a physician. This might explain the discrepancy in prevalence between reported physician‐diagnosed and current AR in our study. Another limitation is the lack of data on AR incidence and sensitisation before the age of 8. Nonetheless, the typical onset of AR during the school years renders this factor unlikely to impact the generalisability of our findings.

In conclusion, this study shows the effects of sex and sensitisation timing on the incidence and remission of AR. These findings spotlight the need for future studies investigating the contributions of early sensitisation and environmental influences in driving AR progression across diverse populations.

## AUTHOR CONTRIBUTIONS

**Styliana Vasileiadou:** Formal analysis; writing – original draft; writing – review and editing; data curation; visualization; methodology. **Emma Goksör:** Supervision; writing – review and editing; funding acquisition; methodology; formal analysis; conceptualization. **Göran Wennergren:** Funding acquisition; methodology; writing – review and editing; supervision; conceptualization. **Eva Rönmark:** Conceptualization; investigation; funding acquisition; methodology; writing – review and editing; project administration; supervision. **Linnea Hedman:** Conceptualization; investigation; funding acquisition; methodology; writing – review and editing; project administration; supervision; data curation.

## FUNDING INFORMATION

Financial support was received from the Swedish Heart‐Lung Foundation, the Swedish Asthma‐Allergy Foundation, the Swedish Research Council, the Swedish Foundation for Health Care Science and Allergy Research (Vårdal), Visare Norr, Norrbotten County Council, ALF – the regional agreement between Umeå University and Västerbotten County Council, Umeå University, the Wallström and Sjöblom Foundation, and the Queen Silvia's Children Hospital Research Foundation, as well as ALF – the regional agreement between the University of Gothenburg and Region Västra Götaland. Additional support was provided by ALK‐Abello (Horsholm, Denmark).

## CONFLICT OF INTEREST STATEMENT

The authors declare no conflicts of interest.

### PEER REVIEW

The peer review history for this article is available at https://www.webofscience.com/api/gateway/wos/peer‐review/10.1111/pai.70078.

## Supporting information


Table S1.

Table S2.

Table S3.

Table S4.

Table S5.

Table S6.

Table S7.
