# Supplementary material for: Allergic rhinitis: Incidence and remission from childhood to young adulthood—A prospective study
Source: Pediatr Allergy Immunol. 2025 Apr 2;36(4):e70078. doi: 10.1111/pai.70078 (PMC11963224; doi:10.1111/pai.70078)
Supplement: Supplementary file 1 — Table S1. [file PAI-36-e70078-s003.docx]

**Table S1** Definitions of potential risk factors included in the unadjusted and adjusted analyses. All information about potential risk factors was obtained from the questionnaire completed by the parents when the child was 8 years old.

| Risk factors | Definition |
| --- | --- |
| Family history of allergic rhinitis | Mother or father with a history allergic rhinitis |
| Short breastfeeding (<4 months) | Breastfeeding less than 4 months |
| Maternal smoking in pregnancy | Did the mother smoke during pregnancy? |
| Parental smoking the first year of life | Mother and/or father smoked during the first year of life |
| Rural (urban) living | Reported rural (urban) living during the first year of life |
| Ever living on a farm | Past or present living on a farm |
| Heavy traffic road close to home | Past or present large busy road or very frequented bus stop within 200 m of the home |
| Dampness at home | Past or present indoor moisture damage or moulds |
| Ever cat (dog) at home | Previously or presently having cat (dog) at home |
| Parental smoking | Presently smoking mother and/or father |
| Fish less than about once per week | How often does the child eat fish?  The possible answers were: at least 3 times per week, almost 2 times per week, almost 1 time per week, almost 1-3 times per month, less than 1 time per month, never. |
| Fruit less than every day | How often does the child eat any kind of fruit?  The possible answers were: Every day at least 2, every day at least one, almost every day, 1-3 times per week, less than 1 time per week. |
| Fast food at least once a week | How often does the child eat fast food?  The possible answers were: Almost 1 time every day, almost every other day, almost 2 times per week, almost 1 time per week, a few times per month, never or almost never. |
| Socioeconomic status | The question was about which profession the adults in the household have and that was coded according to the SEI system. The possible alternatives were manual workers, unemployed, non-manual employees, self-employed, professionals and executives. |
| Eczema at age 8 years | Has the child been diagnosed with eczema by a physician? |
| Asthma at age 8 years | Has the child been diagnosed with asthma by a physician? |
| Food allergy at age 8 years | Is the child allergic to anything in food? The possible answers included the basic food allergens: milk, egg, fish, seafood, wheat, soy, peanut, nuts and almond. |

†SEI, socioeconomic index
