# Supplementary material for: Allergic rhinitis: Incidence and remission from childhood to young adulthood—A prospective study
Source: Pediatr Allergy Immunol. 2025 Apr 2;36(4):e70078. doi: 10.1111/pai.70078 (PMC11963224; doi:10.1111/pai.70078)
Supplement: Supplementary file 2 — Table S2. [file PAI-36-e70078-s002.docx]

**Table S2** Questions on allergic rhinitis administered to participants at ages 8 and 19 years.

| **Questions** |
| --- |
| 1. Has your child ever had a problem with sneezing, or a runny, or a blocked nose when he/she did not have a cold? 2. In the last 12 months, has your child had a problem with sneezing, or a runny, or a blocked nose when he/she did not have a cold? 3. In the last 12 months, has this nose problem been accompanied by itchy-watery eyes? 4. In the last 12 months, how much did this nose problem interfere with your child's daily activities? 5. How often has your child had to use medication for allergic nose/eye problems in the last 12 months? 6. Has your child ever had ‘’hay fever’’? 7. Has your child been diagnosed by a physician as having hay fever or allergic nose/eye problems? |
