# Supplementary material for: Allergic rhinitis: Incidence and remission from childhood to young adulthood—A prospective study
Source: Pediatr Allergy Immunol. 2025 Apr 2;36(4):e70078. doi: 10.1111/pai.70078 (PMC11963224; doi:10.1111/pai.70078)
Supplement: Supplementary file 3 — Table S3. [file PAI-36-e70078-s001.docx]

**Table S3** Characteristics of the 2,250 responders at 19 years of age compared to data provided by non-responders (n=335) when they took part at 8 years of age. Statistical significance is indicated in bold.

|  | Responders | Non-responders | p-value |  |  |
| --- | --- | --- | --- | --- | --- |
| Characteristics | | % (n) | % (n) |  |  |
|  | | 87 (2,250) | 13 (335) |  |  |
| Female sex | | 48.8 (1,097/2,250) | 45.7 (153/335) | 0.319 |  |
| Current AR | | 15.6 (350/2,250) | 16.7 (56/335) | 0.574 |  |
| **Family history of AR** | | **43.9 (987/2,250)** | **37.0 (124/335)** | **0.018** |  |
| **Parental smoking the first year of life** | | **18.2 (401/2,199)** | **29.4 (93/316)** | **<0.001** | |
| Fast food at least once a week | | 10.5 (236/2,239) | 13.2 (44/333) | 0.157 | |
| **Socioeconomic status** | |  |  |  | |
| **Manual workers** | | **25 (517/2,065)** | **34.8 (87/250)** | **<0.001** | |
| **Unemployed** | | **1.4 (28)** | **4.8 (12)** |  | |
| **Non-manual employees** | | **44.0 (908)** | **34.0 (85)** |  | |
| **Self-employed** | | **6.3 (130)** | **6.4 (16)** |  | |
| **Professionals and executives** | | **23.3 (482)** | **20.0 (50)** |  | |
| Eczema at age 8 years | | 15.4 (346/2,250) | 14.3 (48/335) | 0.684 | |
| Asthma at age 8 years | | 7.1 (160/2,250) | 9.3 (31/335) | 0.178 | |
| Sensitisation at age <8 years | | 30.6 (445/1,455) | 25.6 (61/238) | 0.127 | |
| *The figures vary because of missing values. We present the actual percentages out of the number of responses received, not the total cohort.*  † AR, allergic rhinitis | | | | | |
