# Supplementary material for: Allergic rhinitis: Incidence and remission from childhood to young adulthood—A prospective study
Source: Pediatr Allergy Immunol. 2025 Apr 2;36(4):e70078. doi: 10.1111/pai.70078 (PMC11963224; doi:10.1111/pai.70078)
Supplement: Supplementary file 4 — Table S4. [file PAI-36-e70078-s007.docx]

**Table S4** Factors associated with the incidence of allergic rhinitis from age 8 to 19 years, analysed by logistic regression and expressed as adjusted odds ratios with 95% confidence intervals. The analysis was also adjusted for parental socioeconomic status. Statistical significance is indicated in bold.

| Factors | aOR | 95% CI |
| --- | --- | --- |
| Sensitisation |  |  |
| No sensitisation | 1 | Ref. |
| **Sensitisation at age <8 years** | **3.75** | **2.68-5.23** |
| **Sensitization at age >8 years** | **2.57** | **1.82-3.63** |
| **Female sex** | **1.71** | **1.30-2.26** |
| Fast food at least once a week | 1.53 | 0.99-2.36 |
| Asthma at age 8 years | 1.21 | 0.66-2.23 |
| Eczema at age 8 years | 1.22 | 0.82-1.83 |
| Food allergy at age 8 years | 1.18 | 0.78-1.79 |
| Family history of AR | 1.05 | 0.80-1.39 |
| Parental smoking the first year of life | 0.91 | 0.62-1.34 |

† aOR, adjusted odds ratio

‡ 95% CI, 95% confidence interval

§ AR, allergic rhinitis
