# Supplementary material for: Allergic rhinitis: Incidence and remission from childhood to young adulthood—A prospective study
Source: Pediatr Allergy Immunol. 2025 Apr 2;36(4):e70078. doi: 10.1111/pai.70078 (PMC11963224; doi:10.1111/pai.70078)
Supplement: Supplementary file 6 — Table S6. [file PAI-36-e70078-s005.docx]

**Table S****6** Remission rates of allergic rhinitis between 8 and 19 years of age, with or without possible risk factors, using unadjusted odds ratios with 95% confidence intervals to assess the association of each factor.

| Factors | Remission of AR % (n=140) | p-value | OR | 95% CI |
| --- | --- | --- | --- | --- |
| Family history of AR |  |  |  |  |
| No | 40.3 (48/119) |  | 1 | Ref. |
| Yes | 39.8 (92/231) | 1.000 | 0.98 | 0.62-1.54 |
| Short breastfeeding (<4 months) |  |  |  |  |
| No | 31.0 (13/42) |  | 1 | Ref. |
| Yes | 40.8 (122/299) | 0.242 | 1.54 | 0.77-3.08 |
| Maternal smoking in pregnancy |  |  |  |  |
| No | 39.9 (122/306) |  | 1 | Ref. |
| Yes | 36.8 (14/38) | 0.861 | 0.88 | 0.44-1.77 |
| Parental smoking the first year of life |  |  |  |  |
| No | 38.8 (109/281) |  | 1 | Ref. |
| Yes | 44.4 (28/63) | 0.791 | 1.26 | 0.73-2.19 |
| Number of siblings |  |  |  |  |
| 0 | 40.0 (12/30) | 0.865 | 1 | Ref. |
| 1 | 41.2 (73/177) |  | 1.05 | 0.48-2.32 |
| 2 | 34.5 (29/84) |  | 0.79 | 0.34-1.87 |
| >3 | 42.6 (23/54) |  | 1.11 | 0.45-2.76 |
| Area of living the first year of life |  |  |  |  |
| Urban | 38.7 (84/217) | 0.548 | 1 | Ref. |
| Rural | 42.6 (46/108) |  | 0.85 | 0.53-1.36 |
| Ever living on a farm |  |  |  |  |
| No | 40.2 (137/341) | 0.652 | 1 | Ref. |
| Yes | 20.0 (1/5) |  | 0.37 | 0.04-3.37 |
| Heavy traffic road close to home |  |  |  |  |
| No | 41.4 (77/186) |  | 1 | Ref. |
| Yes | 39.5 (62/157) | 0.742 | 0.92 | 0.60-1.43 |
| Dampness at home |  |  |  |  |
| No | 41.7 (93/223) |  | 1 | Ref. |
| Yes | 38.5 (45/117) | 0.062 | 0.49 | 0.24-1.01 |
| Ever dog at home |  |  |  |  |
| No | 39.5 (94/238) |  | 1 | Ref. |
| Yes | 41.3 (43/104) | 0.811 | 1.08 | 0.68-1.73 |
| Parental smoking |  |  |  |  |
| No | 39.6 (106/268) |  | 1 | Ref. |
| Yes | 41.3 (31/75) | 0.791 | 1.08 | 0.64-1.81 |
| Fish less than once per week |  |  |  |  |
| No | 39.4 (109/277) |  | 1 | Ref. |
| Yes | 40.6 (28/69) | 0.891 | 1.05 | 0.62-1.80 |
| Fruit less than every day |  |  |  |  |
| No | 40.4 (107/265) |  | 1 | Ref. |
| Yes | 37.8 (31/82) | 0.701 | 0.90 | 0.54-1.49 |
| Fast food at least once a week |  |  |  |  |
| No | 39.0 (122/313) |  | 1 | Ref. |
| Yes | 45.7 (16/35) | 0.469 | 1.32 | 0.65-2.66 |
| Eczema at age 8 years |  |  |  |  |
| No | 42.3 (101/239) |  | 1 | Ref. |
| Yes | 35.1 (39/111) | 0.791 | 0.74 | 0.46-1.18 |
| Asthma at age 8 years |  |  |  |  |
| No | 39.8 (111/279) |  | 1 | Ref. |
| Yes | 40.8 (29/71) | 0.402 | 1.05 | 0.62-1.78 |
| Food allergy at age 8 years |  |  |  |  |
| No | 43.2 (108/250) |  | 1 | Ref. |
| Yes | 32.0 (32/100) | 0.055 | 0.62 | 0.38-1.01 |

† OR, odds ratio

‡ 95% CI, 95% confidence interval

§ AR, allergic rhinitis
