# Supplementary material for: Allergic rhinitis: Incidence and remission from childhood to young adulthood—A prospective study
Source: Pediatr Allergy Immunol. 2025 Apr 2;36(4):e70078. doi: 10.1111/pai.70078 (PMC11963224; doi:10.1111/pai.70078)
Supplement: Supplementary file 7 — Table S7. [file PAI-36-e70078-s004.docx]

**Table S7** Factors associated with remission of allergic rhinitis from age 8 to 19 years, analysed by logistic regression and expressed as adjusted odds ratios with 95% confidence intervals. The analysis was also adjusted for parental socioeconomic status. Statistical significance is indicated in bold.

| Factors | aOR | 95% CI |
| --- | --- | --- |
| Sensitisation |  |  |
| No sensitisation | 1 | Ref. |
| **Sensitisation at age <8 years** | **0.26** | **0.13-0.53** |
| **Sensitisation at age >8 years** | **0.20** | **0.05-0.77** |
| Female sex | 0.59 | 0.31-1.11 |
| Family history of AR | 0.81 | 0.42-1.53 |
| Ever cat at home | 1.01 | 0.44-2.31 |

† aOR, adjusted odds ratio

‡ 95% CI, 95% confidence interval

§ AR, allergic rhinitis
